# Comparison of Fracture Behavior in Single-Edge Notched Beams Reinforced with Steel Bars or CFRP Bars

**DOI:** 10.3390/ma17102216

**Published:** 2024-05-08

**Authors:** Zhiyong Wang, Yushu Yang, Min Song, Jie Zhang, Zhihua Wang

**Affiliations:** 1 College of Mechanical and Vehicle Engineering, Taiyuan University of Technology, Taiyuan 030024, China; yangyushu163@163.com (Y.Y.); songmin595@163.com (M.S.); zhangjie04@tyut.edu.cn (J.Z.); wangzh077@163.com (Z.W.); 2 Institute of Applied Mechanics, Taiyuan University of Technology, Taiyuan 030024, China; 3 Shanxi Transportation Technology R&D Co., Ltd., Bridge and Tunnel Engineering Research Institute, Taiyuan 030000, China; 4 Department of Civil and Environmental Engineering, National University of Singapore, 1 Engineering Drive 2, Singapore 117576, Singapore

**Keywords:** notched concrete beam, CFRP bars, steel bars, three-point bending test, failure pattern

## Abstract

To explore and compare the failure modes, deformation behaviors, and load-bearing capacities of single-edge notched (SEN) beams strengthened with carbon fiber-reinforced polymer (CFRP) and steel bars, static and dynamic three-point bending tests on both types of concrete beams have been carried out in this study. During the static tests, the electro-hydraulic servo machine served as a loading device to apply pressure to CFRP beams and reinforced concrete (RC) beams. During the impact experiments, different impact velocities were imparted by adjusting the drop hammer’s height. Thus, information regarding crack propagation, energy absorption, and deformation was obtained. The results from the static tests showed that the RC beams predominantly experienced shear failure. In contrast, the CFRP beams primarily exhibited bending–shear failure, attributed to the relatively weaker bond strength between the bars and the concrete. Impact tests were conducted at three different velocities in this study. As the impact velocity increased, both types of concrete beams transitioned from bending failure to bending–shear failure. At the lowest velocity, the difference in energy absorption between beams reinforced with different materials was insignificant during the bending process. However, at the highest velocity, CFRP beams absorbed less energy than RC beams. The study of structures’ impact failure modes and their mechanical characteristics offers valuable references for the anti-collision design and protection of structures.

## 1. Introduction

The reinforced concrete (RC) beam, which incorporates steel reinforcement within a concrete matrix, is an essential component in modern architecture and bridge construction. Its performance directly impacts the safety, stability, and durability of the structures they uphold. It combines the strong compression resistance of concrete with the flexibility of steel, making the RC beam more resilient and durable than the concrete beam. Therefore, the failure and degradation mechanisms of RC beams has been a subject of considerable interest to researchers and engineers [1,2,3,4]. Numerous studies have extensively explored the behavior of RC beams under various loading conditions [5,6,7,8,9,10,11,12,13,14]. They have studied how factors like impact velocity, reinforcement arrangement, and concrete strength influence their impact failure patterns. However, the reinforcing steel is prone to corrosion in damp or salty environments. Issues like inadequate concrete cover, poor design or construction practices, improper concrete mixes [15], and corrosive conditions can compromise concrete’s protective role [16,17], increasing the risk of reinforcement corrosion [18,19]. As corrosion begins, structural integrity gradually deteriorates over time. With the development of materials science, carbon fiber-reinforced polymer (CFRP) is expected to become a substitute for traditional reinforcing steel. This is because CFRP offers higher tensile strength and better corrosion resistance [20,21,22]. Recent research indicates that CFRP beams and RC beams show similar failure patterns. However, CFRP beams usually experience greater bending [23] and exhibit a weaker bond with concrete [24]. To better understand the behavior of these concrete beams in different situations, researchers have studied their failure mechanisms in detail [25,26,27]. This has led to the formulation of applicable design standards, informed by experiments conducted under static loads [28,29,30].

Although valuable insights have been gained from previous research, much focus has been placed on the mechanical behavior of intact beams. However, this focus may not fully capture the complex failure mechanisms under different loads. Pre-casting notches in concrete beam specimens is a widely used method to clarify failure modes and internal crack evolution. This approach not only simulates potential cracking and fracturing scenarios, but also deepens the understanding of how crack propagation affects structural integrity. It enables a more precise evaluation of concrete beam performance and failure mechanisms under loads. Several studies have investigated the static fracture toughness and the distribution of localized fracture energy in SEN beams reinforced with steel [31,32]. Mi et al. [33] proposed an analysis method that assumes an elastoplastic constitutive relationship for the behavior of reinforcing steel. This method utilizes steel strain to calculate tensile stress and provides further insight into the mechanical behavior of cracks in RC beams. This approach has been extensively applied in various fracture models to investigate the role of reinforcement in concrete beam fracture behavior. Alrayes et al. [34] introduced a numerical modeling technique that employs the scaled boundary finite element model (SBFEM) for the simulation of crack propagation, which is applied to simulate the three-point bending tests of SEN concrete beams under various loading scenarios, confirming the efficacy of the proposed model. Additionally, alongside the three-point bending test, the four-point bending test is highly regarded for the study of beam bending failure behavior due to its ability to maintain a constant bending moment at specific locations on the beam, thereby achieving zero shear. However, in the context of impact testing, the use of a four-point bending setup might introduce additional complexities in the actual loading conditions, complicating the interpretation of results. Therefore, the three-point bending test is more widely used due to its simplicity and its ability to provide better control over the experimental process.

In previous studies, Song [34] conducted impact three-point bending tests on a plain concrete beam and a lightly reinforced concrete beam. This study assessed the influence of steel reinforcement on the crack velocity, fracture toughness, and fracture energy. To investigate the properties of SEN beams reinforced with CFRP bars, the present study conducts detailed experimental analyses on SEN beams reinforced with either CFRP or steel bars. This exploration aims to elucidate the effects of these reinforcements on concrete beams subjected to both impact and static loading conditions. The objective of this study is to further examine the failure modes of these beams and to address specific gaps in existing knowledge.

This document presents three-point bending tests on SEN beams reinforced with either CFRP or steel under static loads. It utilizes high-precision data acquisition and photographic technologies to accurately record the strain reactions of reinforcing steel and the initiation and propagation of cracks under applied loads. This provides comprehensive information for strain field analysis. Additionally, impact experiments were conducted, varying the drop height of a hammer to represent different impact velocities. High-speed imaging and data collection technologies were employed to precisely record the impact force, beam response force, mid-span displacement, crack propagation paths, and strain responses in both CFRP and RC beams. This contributes to a comprehensive analysis of how impact velocity influences the performance of each beam type. The results from impact testing reveal the effects of different impact velocities on beam mechanical performance. This highlights the differences in deformation behaviors and energy absorption capabilities between CFRP and RC beams under impact stress. Further analysis of the deformation patterns, tensile forces in reinforcement, and crack failure modes of the concrete beams provides a comprehensive framework for engineering design and evaluation considerations.

## 2. Experimental Procedure

### 2.1. Specimen and Mechanical Properties of Materials

**Figure 1:**
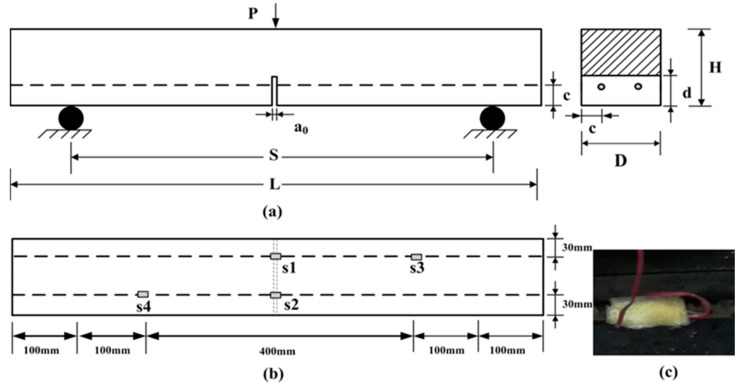
(**a**) A schematic diagram of the SEN beam; (**b**) the distribution of strain gauges inside the specimen; (**c**) the corresponding protection.

The concrete beams applied in this investigation were reinforced with either two CFRP bars or two steel bars. The tensile tests for three CFRP bars are conducted using an electro-hydraulic servo machine, purchased from Shenzhen Wance Testing Machine Co., Ltd. (Building 3, Yinjin Science and Technology Industrial Park, Fengjing South Road, Guangming District, Shenzhen, Guangdong, China), according to the GB/T30022-2013 [35] test method. The tensile strength ($f_{u}^{cfrp}$) and elastic modulus ($E_{cfrp}$) of the 12 mm longitudinal CFRP bars are 2033 MPa and 161 GPa, respectively. The CFRP bars was purchased from Nanjing Hitech Composites Co., Ltd. (No. 26 Chaoyang Road, Dongping Industrial Zone, Lishui District, Nanjing, Jiangsu, China). The tensile strength ($f_{u}^{s}$) and elastic modulus ($E_{s}$) of the steel bars are 450 MPa and 200 GPa, respectively, provided by Taiyuan Xinjunchuang Trade Co., Ltd. (No. B53, North Market of Qitianrui Steel Market, Xinghua West Street, Taiyuan, Shanxi, China). The maximum size of aggregates is 15 mm, and the mixing proportion (cement:water:coarse aggregate:sand) by weight is 1:0.35:2.62:1.55. Cement with a strength grade of P.O 42.5, purchased from Taiyuan Lionhead Cement Co., Ltd. (No. 1, Kaicheng Street, Wanbolin District, Taiyuan, Shanxi, China), is applied during the specimen preparation process. The mean compressive strength ($f_{cu}$) and mean split tensile strength ($f_{ts}$) of the concrete at 28 days of curing are 63.79 MPa and 3.15 MPa, respectively. The notch is created by the concrete pouring process and is of a rectangular shape. All the specimens had rectangular cross sections with a notch depth (*D*) of 150 mm, notch height (*d*) of 45 mm, notch width ($a_{0}$) of 4 mm, specimen height (*H*) of 150 mm, specimen length (*L*) of 0.8 m, and clear span length (*S*) of 0.6 m. Additionally, the distance from the side to the fulcrum is measured as 100 mm. A schematic of these specimens is presented in Figure 1a. In the figure, the left side presents the front view of the beam, while the right side displays the cross-sectional view at the mid-span. The dashed lines and small dots in the figure indicate the presence of CFRP or steel reinforcement. A schematic of these specimens is presented in Figure 1a. The strain gauges (model: BE120-3AA), purchased from Avic Zhonghang Electronic Measuring Instruments Co., Ltd. (No. 166, West Avenue, High-tech Zone, Xi’an, Shaanxi, China), are attached to the bars to monitor the deformation, and the locations are illustrated in Figure 1b, which provides a top view of the specimen. The strains at different locations of the steel bars are described as s1, s2, s3, and s4, and the strains at the same locations for the CFRP bars are presented as c1, c2, c3, and c4, respectively. Notably, the strain gauges located at c1–c4 and s1–s4 are attached to the underside of the bar, along the direction of the reinforcement bar, to measure tensile strain. To avoid damage to the strain gauges during the concrete casting process, the strain gauges are wrapped with gauze bonded by epoxy resin purchased from Anhui Hengyuan Chemucol Co., Ltd. (No. 16, Zijin Road, Circular Economy Park, Huizhou District, Huangshan, Anhui, China), as shown in Figure 1c. Prior to the concrete pouring process, a plexiglass sheet is positioned at the mid-span of the beam mold, corresponding to the intended notch location in the finished concrete beam, to support the steel or CFRP bars and to pre-form the notch shape. Given the plexiglass’s tight integration with the concrete during the advanced stages of curing and its negligible impact on subsequent fracture behavior analysis, it is retained.

### 2.2. Three-Point Bending Test

**Figure 2:**
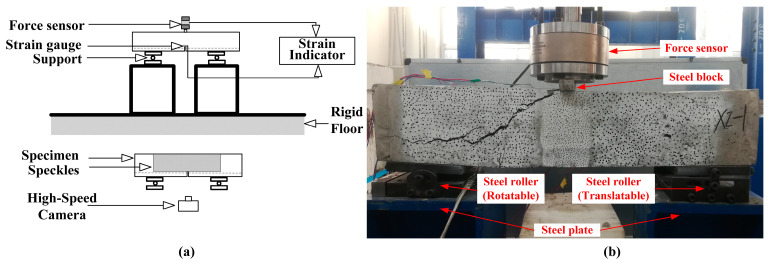
A diagram of the static testing configuration; (**a**) the impact three-point bending tests setup schematic; (**b**) an operational photo of the three-point bending test.

To examine how SEN beams respond structurally when reinforced with either CFRP bars or steel bars, we conducted three-point bending tests. These tests were carried out using a self-balancing counter-force frame testing machine provided by the College of Civil Engineering, Qinghai University (No. 251, Ningda Road, Xining, Qinghai, China). To apply the load, we used a 300 kN hydraulic actuator anchored to a steel frame. Under static loads, the beams were placed on two steel supports. One end was secured by a fixed support, and a roller support was positioned at the other end. The specimens were tested under displacement-controlled loading, with a loading rate set at 1 mm/min, until the concrete beams were destroyed. During the loading process, we used a high-speed data acquisition system (DH3818), provided by DongHua Testing Technology Co., Ltd. (No. 208, Xingang Avenue, Jingjiang, Jiangsu, China), to monitor the applied load and the strain on the bars. Data were collected at a rate of 1 Hz. Furthermore, the fracture process of specimens with speckled surfaces was captured by a camera. Subsequently, the images of the specimens were processed to derive the strain field of the beam surface. The static test device and data acquisition equipment are depicted in Figure 2.

**Figure 3:**
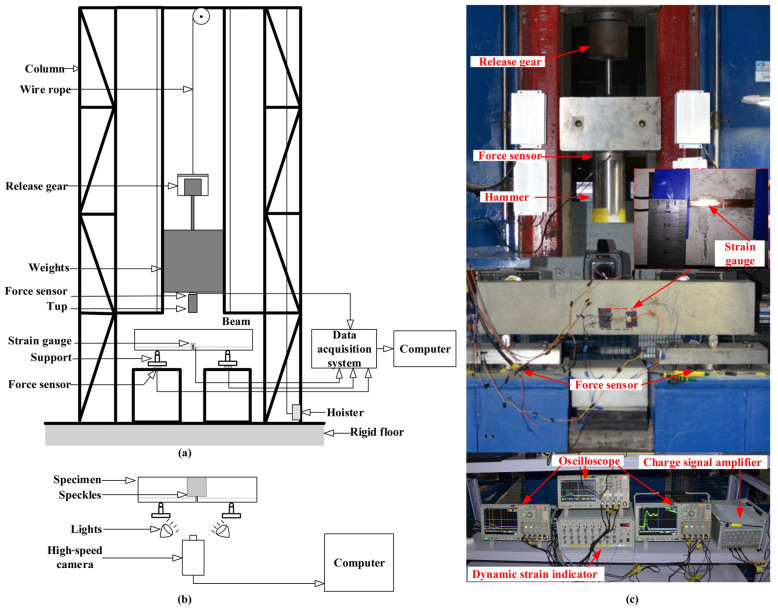
The schematic (**a**,**b**) and an actual snapshot (**c**) of the experimental setup for the impact three-point bending test.

The impact three-point bending tests were conducted using a drop-hammer impact device designed by Qinghai University. The experimental setup is shown in Figure 3. The hammer weight was 106.05 kg, and three drop heights (H=50 mm, c, and 250 mm) were adopted. Thus, three impact velocities were applied in this study: $9.89\times 10^{2}\;\mathrm{mm}/s$, $1.71\times 10^{3}\;\mathrm{mm}/s$, and $2.21\times 10^{3}\;\mathrm{mm}/s$. The corresponding concrete beams reinforced with steel bars were marked as S2-50, S2-150, and S2-250, respectively. Similarly, the specimens with CFRP bars were marked as F2-50, F2-150, and F2-250, respectively.

To prevent stress concentration when the hammer head contacts the beam, a steel block is positioned at the center of the specimen’s top surface. Before impact testing, three force sensors are affixed to the hammer and supports to measure impact force and reaction forces, as shown in Figure 3. A high-speed camera (sampling rate = 50 kHz) captures the impact process and records mid-span beam deflection. Surface speckle coordinates are analyzed in order to study strain field evolution. Data under varying impact velocities are collected with three oscilloscopes at a 2 MHz sampling rate. All oscilloscopes synchronize with the impact force signal, while the high-speed camera operates in manual trigger mode.

## 3. Experimental Results and Discussion

### 3.1. Quasi-Static Loadings

#### 3.1.1. Failure Pattern

After conducting static three-point bending tests on SEN beams with various reinforcement bars, the bending bearing capacity for each was determined ($P_{u}^{5}=32.72\;\mathrm{kN}$, $P_{u}^{cfrp}=64.8\;\mathrm{kN}$). The static shear capacity for specimens is calculated using a conventional prediction equation, Equation (1) [36]:

(1) $$V_{u}=1.75f_{t}Dh_{0}/(\lambda +1)$$

where $f_{t}$ is the concrete’s tensile strength ($f_{t}=3.15\;\mathrm{MPa}$), $h_{0}$ is the effective height of the beam section ($h_{0}=105\;\mathrm{mm}$), and λ is the shear–span ratio (λ=2.86). The static shear capacities for specimens are obtained ($V_{u}=22.49\;\mathrm{kN}$). The static bending–shear capacity ratio is obtained with Equation (2).

(2) $$\alpha =V_{u}/P_{u}$$

The static bending–shear capacity ratios for concrete beams with CFRP bars and steel bars are 0.69 and 0.35, respectively. According to the conclusion from Kishi [37], the concrete beams in this study should collapse in a shear failure mode under static loads because their ratios are smaller than 1.0.

**Figure 4:**
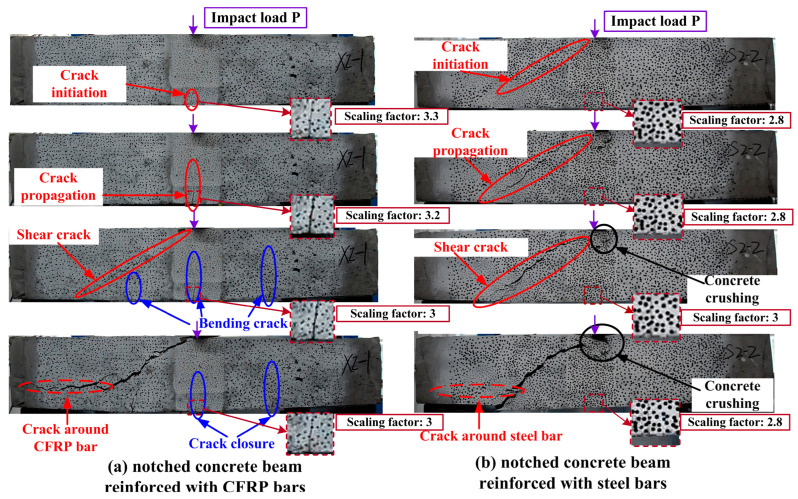
Failure process of SEN beams reinforced with (**a**) CFRP bars and (**b**) steel bars under static loads.

The static failure process is captured by a camera, as shown in Figure 4. The conditions of crack propagation, the locations of notches, and the impact load P are indicated in the figure, accompanied by the scaling factors for each sub-figure. During the initial loading stages of CFRP beams, bending cracks initiate from the notch root. With the progression of applied load, shear cracks appear and gradually propagate in the beams. After reaching a certain level of deformation, the cracks extend along the direction of the CFRP bar, causing bending–shear failure to occur in the CFRP beams. In RC beams, as loading increases, shear cracks propagate towards the right support, concomitant with the emergence of micro-cracks in the concrete surrounding the steel bars. Subsequently, bending failure occurs. Compared to the strong bond observed between steel bars and concrete, the bond between CFRP bars and concrete is relatively weaker, thereby resulting in distinct failure modes between the two types of beams.

#### 3.1.2. Quasi-Static Response

**Figure 5:**
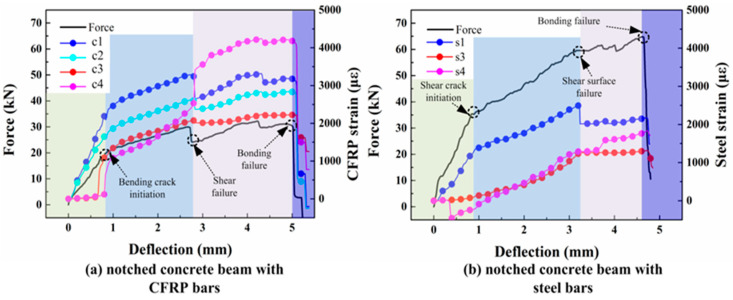
Under static loading, the failure process and stress distribution at different locations on CFRP beams and reinforced concrete beams.

Figure 5 presents the curves plotting the load (captured by the force sensor) and the strain of the bars versus the mid-span deflection. The deflection, oriented in the longitudinal direction of each specimen, was captured by a high-speed camera. The strains at different locations of the CFRP bars are marked as c1, c2, c3, and c4, respectively. Similarly, the strains of the steel bars are presented as s1, s2, s3, and s4, respectively. Some test data of steel strain at the midpoint (s2) was lost due to malfunction of the data acquisition system. It can be seen that both of the load–mid-span deflection curves for the SEN beams with CFRP bars or steel bars can be divided into four stages, highlighted with four different background colors. From the first stage to the fourth stage, they are respectively highlighted in yellow, blue, pink, and purple. As presented in Figure 5a, at the first stage, the load and strains of c1 increase gradually, which results from the concrete beam still being in the elastic stage. In the second stage, as bending cracks are initiated, the growth trend of the applied load and the strains of c1 and c2 weaken. Meanwhile, the strains at both sides of the CFRP bars (c3/c4) increase sharply, indicating deformation of the CFRP bars. Notably, their increasing speeds are almost the same, indicating stable crack propagation. With the increase in mid-span deflection, shear cracks become predominant and propagate, causing a decrease in strains at the midpoint of the CFRP bars and in the applied load. This phenomenon corresponds to the light blue area in Figure 5a. As the mid-span deflection increases, shear cracks begin to propagate, leading to a decrease in strains at the midpoint of the CFRP bars and in the applied load. Both the RC beams and the CFRP beams exclusively exhibit shear cracking. As the shear crack is initiated, the increasing trend of steel strain at the midpoint (s1) weakens, while the steel strains at other locations (s3/s4) remain almost unchanged, as described in Figure 5b. This suggests that the strong bonding effect of steel bars suppresses the initiation of bending cracks at mid-span. During the third stage, the strains of CFRP bars and their loads continue to increase until the beam fails. As the bond between the CFRP bars and the concrete is ruined, separation between the concrete and the CFRP bars appears. Finally, the beam fractures into two parts along the shear surface. Similar phenomena are also present in the fracture process of RC beams.

The static test results are presented in Table 1. Due to the lower elastic modulus of CFRP bars, CFRP beams exhibit lower ductility and wider cracks compared to RC beams [38,39,40]. Therefore, under the same reinforcement ratio, RC beams have a higher static bending capacity than CFRP beams. Additionally, CFRP beams are more prone to bending cracks due to their weaker bond strength [41].

### 3.2. Impact Test Results

#### 3.2.1. Failure Pattern

**Figure 6:**
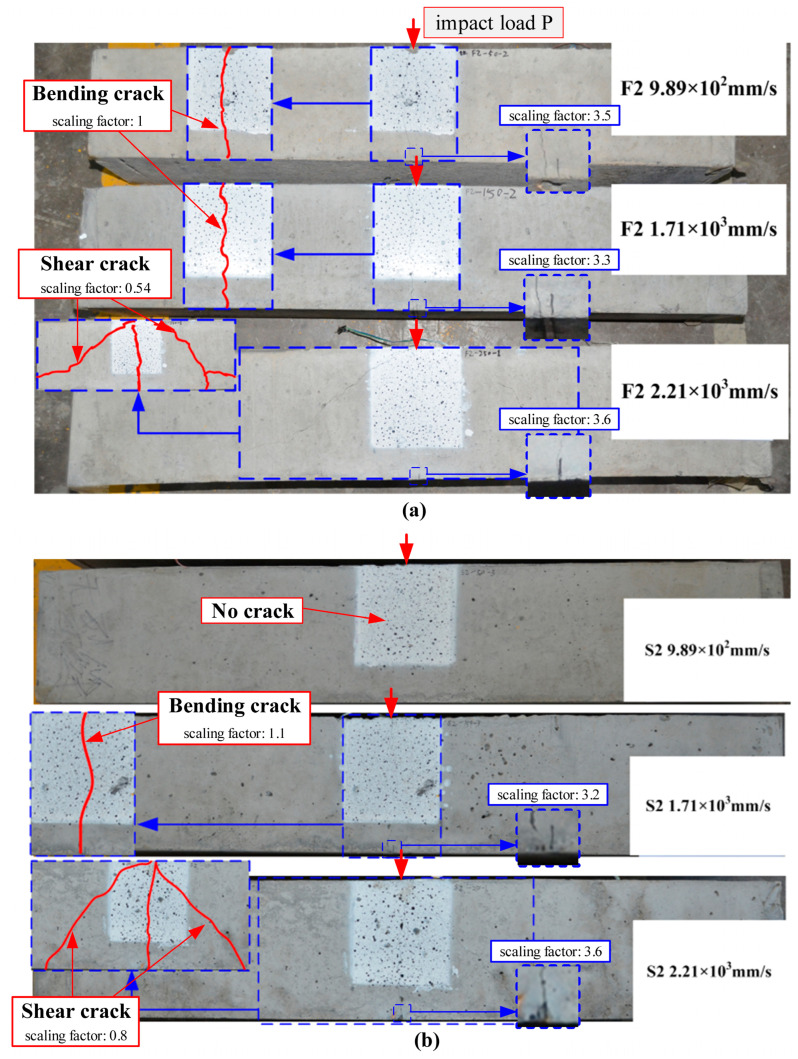
Images of the notches and failure modes of SEN beams reinforced with (**a**) CFRP bars and (**b**) steel bars.

To investigate the impact failure patterns of SEN beams reinforced with CFRP bars or steel bars, three impact velocities were applied. The crack propagation and the notches of each specimen are presented in Figure 6, where the scaling factor is explicitly marked within the images. Due to the inadvertent coverage of the notch’s front face by a small quantity of concrete during the pouring process of the $S2\;9.89\times 10^{2}\;\mathrm{mm}/s$ specimen, the notch is not discernible in the photograph. Nonetheless, this minor concrete coverage exerts a negligible influence on the crack propagation; therefore, it has been disregarded in the analysis. From these observations of crack patterns, CFRP beams collapse in a bending failure when the impact velocity is less than or equal to $1.71\times 10^{3}\;\mathrm{mm}/s$. As the hammer separates from the steel block, a part of the elastic deformation of CFRP bars recovers, which causes the closure of cracks, as shown in Figure 6a. When the impact velocity is $2.21\times 10^{3}\;\mathrm{mm}/s$, the shear cracks are generated from supports to the loading point, and the bending crack initiated from the notch root is inhibited. This phenomenon indicates that the beams collapse in a bending–shear failure mode. In the RC beams, no crack is generated when $v=9.89\times 10^{2}\;\mathrm{mm}/s$. When the velocity is $1.71\times 10^{3}\;\mathrm{mm}/s$, CFRP beams also experience bending cracks. As the velocity increases to $2.21\times 10^{3}\;\mathrm{mm}/s$, shear cracks similarly emerge. This leads to the conclusion that at lower velocities, both types of beams primarily undergo bending failure. However, this mode transitions to bending–shear failure when the velocity reaches $2.21\times 10^{3}\;\mathrm{mm}/s$.

#### 3.2.2. Impact Response

**Figure 7:**
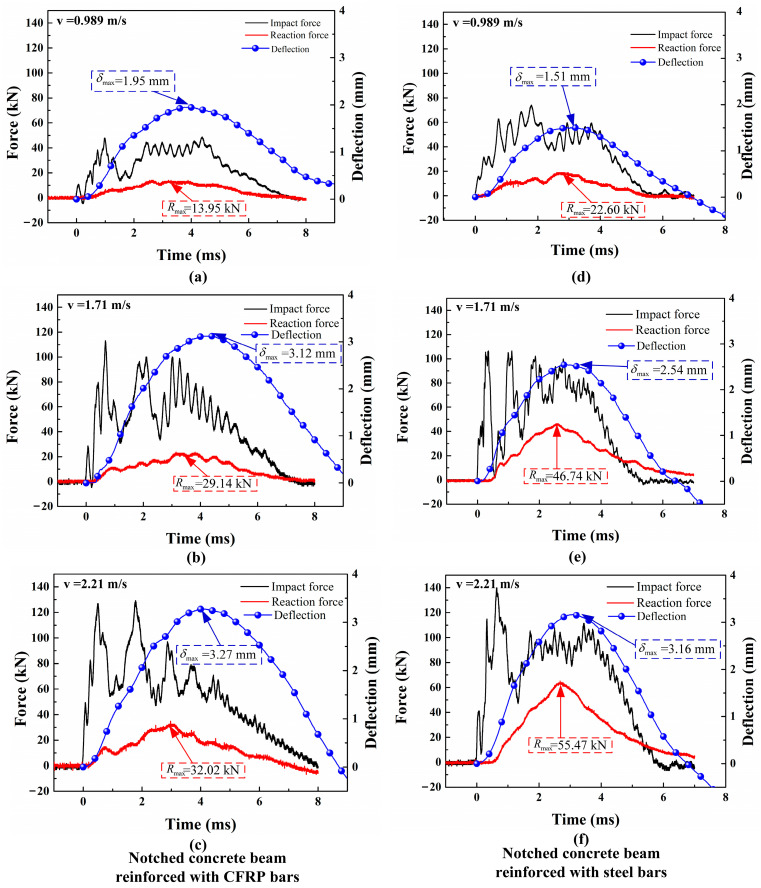
Temporal evolution curves of loads and mid-span deflections for SEN beams reinforced with CFRP bars (**a**–**c**) or steel bars (**d**–**f**) under three impact velocities.

The typical curves of impact force, reaction force, and mid-span deflection over time in SEN beams, reinforced with CFRP bars and steel bars, that are subjected to impact loads are illustrated in Figure 7. It can be observed that the impact response of CFRP beams is similar to that of RC beams. When the hammer contacts the beam, the impact force rapidly increases to its maximum value, while the mid-span deflection and reaction force response are delayed. During the initiation and expansion of cracks, the specimen releases a significant amount of energy, resulting in a reduction of the impact force. Meanwhile, both the deflection and the reaction force gradually increase to their peak values. Subsequently, the impact force, reaction force, and mid-span deflection decrease as the bars’ elasticity recovers. According to the figure, the maximum mid-span deflections for the CFRP beams are 1.95 mm, 3.12 mm, and 3.27 mm under the three different impact velocities; this suggests that beam deformation is increased with rising impact velocity. A similar trend is observed in the impact deflection response of the RC beams, with deflections of 1.51 mm, 2.54 mm, and 3.16 mm, respectively. It is worth noting that the CFRP beams demonstrate greater maximum mid-span deflections compared to the RC beams under different impact velocities. Confirmed by other research, this phenomenon is primarily attributed to the comparatively lower elastic modulus of CFRP bars [23,38].

Compared with the impact forces, the reaction forces present fewer fluctuations and resemble a half-sine wave. Kishi [42] suggested that the reaction forces measured from supports can better reflect the impact resistance than the impact force. Therefore, the sum of reaction forces measured from two supports serves as the impact bearing capacity of the beam, as plotted in Figure 7. It should be noted that the impact bearing capacity of CFRP beams is lower than that of RC beams at the same reinforcement ratio. Therefore, the enhancement of impact bearing capacity by CFRP bars is weaker than that caused by steel bars. Figure 7 illustrates that the maximum reaction forces fall below the impact bearing capacities. This is because although the specimens were not completely destroyed, they did exhibit some bending–shear cracks during the impact process.

**Figure 8:**
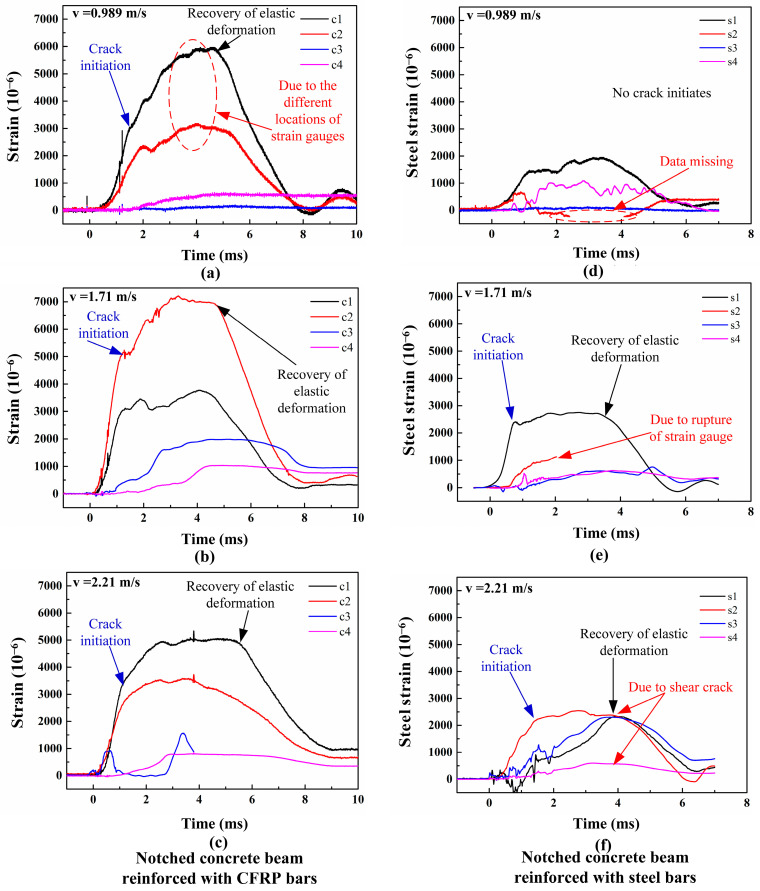
Strain time histories of CFRP bars (**a**–**c**) and steel bars (**d**–**f**) under three impact velocities.

During the impact process, the strains at different locations of CFRP and steel bars were recorded, as shown in Figure 8. The strains at the midpoint of the CFRP beams are labeled as c1 and c2, while the strains at the quarter-span and three-quarter-span positions are labeled as c3 and c4, respectively. Similarly, the strains in the steel bars are identified as s1, s2, s3, and s4, respectively. As depicted in Figure 8a, the strains at the midpoints of the CFRP bars (c1 and c2) show differences. This variation can be elucidated by analyzing the effects of bending deformation. As the mid-span displacement of the beam escalates, the upper surface of the CFRP bar encounters compression, whereas the lower surface is exposed to tension. This phenomenon leads to a marked increase in strain on the underside of the CFRP bar. Although strain gauges are secured directly underneath the bar using gauze and epoxy resin, the CFRP bar may experience slight rotation during the concrete pouring process, potentially altering the position of the strain gauges. Consequently, a discrepancy may exist between the peak strain values at locations c1 and c2.

As the hammer contacts the specimen, the strains at the midpoints of the CFRP bars (c1 and c2) gradually increase. Meanwhile, the strains between the mid-spans and supports of the CFRP bars (c3 and c4) remain constant. Upon the initiation of the bending crack at the notch root, the upward trend of strains at the midpoint begins to weaken. Subsequently, the strains marked as c1 and c2 decrease after reaching their peak values, which is attributed to the elastic deformation recovery in CFRP bars. As depicted in Figure 8a–c, at an impact velocity of $1.71\times 10^{3}\;\mathrm{mm}/s$, the peak strain observed in the CFRP bars at positions c2, c3, and c4 exceeds that recorded at $9.89\times 10^{2}\;\mathrm{mm}/s$. When the impact velocity is $2.21\times 10^{3}\;\mathrm{mm}/s$, the maximum strains at c2, c3, and c4 are smaller than that observed at other impact velocities. Between 2.1 ms and 5.3 ms, the strain at the midpoint of the CFRP bars shows a plateau phase, with a value of approximately 5000. The reason for this phenomenon is that shear failure gradually becomes the dominant factor in the structural response. Consequently, the deformation of the CFRP beams is also limited.

As illustrated in Figure 8d–f, the strain–time curves of steel bars closely adhere to the general trend observed in those of CFRP bars. Moreover, strains at the mid-span positions of steel bars are consistently lower than those of CFRP bars. Notably, the strains at the midpoints of CFRP bars (c1/c2) gradually reach a plateau value after surpassing the crack strain, whereas the strains of steel bars rapidly attain their maximum value, followed by a swift appearance of the strain plateau. This disparity may be attributed to the enhanced bonding effect between steel bars and concrete, a factor warranting further exploration in subsequent research endeavors [43]. In conclusion, by maintaining a constant reinforcement ratio, the deformation of CFRP bars surpasses that of steel bars across various impact velocities.

The constraints imposed by steel bars or CFRP bars can be characterized by a pair of concentrated traction forces acting on the crack face [44,45], with the corresponding equation formulated as Equation (3).

(3) $$F=\frac{nE\varepsilon_{\max}\pi d^{2}}{4}$$

In this equation, *n*, *E*, and *d* represent the quantity (*n* = 2), elastic modulus, and diameter of the bars, respectively. When $v=9.89\times 10^{2}\;\mathrm{mm}/s$, the maximum strains at the midpoints of steel bars and CFRP bars are denoted as 1890 με and 4490 με, respectively. The yield strain of steel bars can be calculated with Equation (4).

(4) $$\varepsilon_{y}^{s}=f_{t}^{s}/E_{s}$$

where $f_{t}^{s}$ and $E_{s}$ are the tensile strength and elastic module of the steel bar, respectively. Hence, the yield strain for steel bars is denoted as 2250 με, while a similar methodology is applied to determine the yield strain of CFRP bars as 12627 με. According to Equation (3), the traction forces exerted by the steel bars and CFRP bars at their mid-spans are denoted as 85.46 kN and 163.43 kN, respectively. Similarly, when impact velocity is $1.71\times 10^{3}\;\mathrm{mm}/s$ and $2.21\times 10^{3}\;\mathrm{mm}/s$, the traction forces given from the steel bars are 101.73 kN and 97.21 kN, respectively, and the traction forces from the CFRP bars are 191.09 kN and 154.69 kN, respectively. Therefore, much more traction force is applied on the crack surface in CFRP beams. Despite the higher traction force exerted on the crack surfaces by CFRP bars at the same reinforcement ratio, CFRP beams demonstrate a lower impact bearing capacity compared to RC beams.

#### 3.2.3. Absorbed Energy

**Figure 9:**
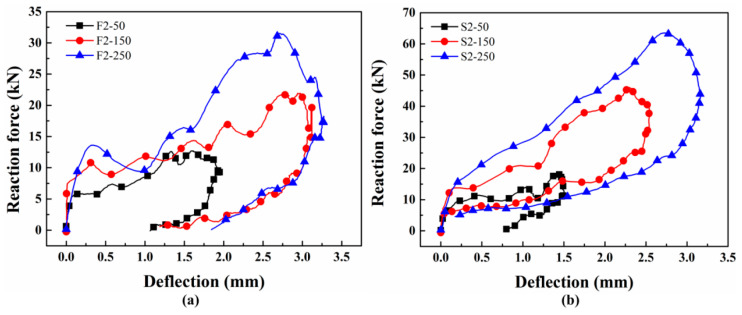
Typical reaction force versus mid-span deflection for SEN beams reinforced with (**a**) CFRP bars or (**b**) steel bars.

Assuming that the reaction force and mid-span deflection are triggered simultaneously [46,47], the reaction force–mid-span deflection curves for the two types of beams under different loads are presented in Figure 9. In Figure 9a, when the impact velocity is $2.21\times 10^{3}\;\mathrm{mm}/s$, the reaction force of a CFRP beam initially increases with the mid-span deflection at the beginning of the impact. With the initiation of the bending crack, the energy stored in the concrete is released, leading to a decrease in the reaction force. The propagation of the crack at mid-span is inhibited by the CFRP bars, and some shear cracks start to generate. Then, the reaction force increases again with the growth of mid-span deflection. With the unloading of the hammer, both the reaction force and mid-span deflection show a decreasing trend, resulting in the reaction force–mid-span deflection curve turning around.

As illustrated in Figure 9b, the response curves of reaction force versus mid-span deflection for RC beams exhibit similarities to those observed for CFRP beams. The decrease in reaction force during the initial loading phase for RC beams is minimal, which may be attributed to the stronger bonding effect between the steel bars and concrete. By integrating these curves, the energy absorbed during the impact failure process of the beams can be calculated. Taking the kinetic energy of the hammer as the input energy, the proportion of absorbed energy is determined. The test results for SEN beams reinforced with different types of bars are presented in Table 2 and Table 3, respectively.

**Figure 10:**
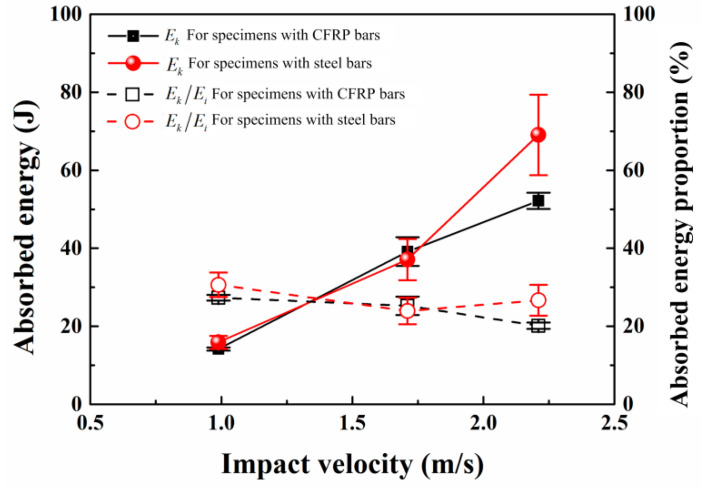
The absorbed energy and its ratio for the specimens under three impact velocities.

As depicted in Figure 10, the graph illustrates both the absorbed energy and its proportion under three impact velocities. With increasing impact velocity, SEN beams exhibit higher energy absorption, regardless of whether they are CFRP beams or RC beams. Notably, for impact velocities less than or equal to $1.71\times 10^{3}\;\mathrm{mm}/s$, the disparity in absorbed energy between the two reinforcement types is minimal. However, at a velocity of $2.21\times 10^{3}\;\mathrm{mm}/s$, the disparity in energy absorption between the two materials increases, correspondingly leading to a widening gap in their energy absorption. This suggests that CFRP beams undergo significant deformation at a velocity of $2.21\times 10^{3}\;\mathrm{mm}/s$, while the strong bond between steel bars and concrete in RC beams prevents significant deformation.

When the velocity is $9.89\times 10^{2}\;\mathrm{mm}/s$ and $1.71\times 10^{3}\;\mathrm{mm}/s$, the difference in energy absorption ratios between the two types of beam is minimal. At an impact velocity of $2.21\times 10^{3}\;\mathrm{mm}/s$, the energy absorption ratio of CFRP beams decreases, while that of RC beams increases. This is attributed to the lower bond strength between CFRP bars and concrete compared to steel bars and concrete. Consequently, when bond-slip occurs between CFRP bars and concrete, the impact bearing capacity of the CFRP beams decreases, resulting in a corresponding reduction in absorbed energy. Conversely, due to the greater bond strength between steel bars and concrete, along with the superior bending and shear resistance of the steel bar, the RC beam absorbs more energy when resisting impact. This characteristic is also evident from the failure modes of the two types of beams. At an impact velocity of $2.21\times 10^{3}\;\mathrm{mm}/s$, the failure mode of both types of beam transitions from bending failure to bending–shear failure. Shear cracks generated in CFRP beams propagate along the direction of the CFRP bars, while shear cracks in steel-reinforced concrete beams propagate towards the supports, showcasing the comparatively weaker bond strength between CFRP bars and concrete compared to steel bars and concrete.

## 4. Conclusions

To investigate the influence of steel and CFRP bars on the failure impacts of SEN beams, both static and impact three-point bending tests were carried out. Subsequently, an analysis discussed the strains of the bars at different locations, failure patterns, and load capacities of SEN beams reinforced with either CFRP or steel bars. The key findings are summarized as follows:

(i) Under static loads, SEN beams reinforced with steel bars primarily exhibit shear failure, whereas beams reinforced with CFRP bars demonstrate a bending–shear failure mode. Under impact conditions, with increasing impact velocity, the failure mode transitions from bending to bending–shear failure.
(ii) In this study, SEN beams reinforced with CFRP bars showed lower static and impact carrying capacities compared to RC beams. Additionally, under both static and impact conditions, CFRP beams exhibit weaker deformation resistance compared to RC beams. As the impact velocity increases, the absorbed energy for CFRP beams or RC beams increases in the bending failure pattern. When shear failure occurs, CFRP beams consume less energy compared to RC beams, which is attributed to the weaker bonding between CFRP bars and concrete.

## Figures and Tables

**Table 1:** Quasi-static test results.

Stage	Specimen Data	SEN Beam Reinforced with CFRP Bars	SEN Beam Reinforced with Steel Bars
Initiation of bending crack	Crack load/kN	20.18	35.13
	Strain at the midpoint of the bar/με	2267.53/1798.75	1317.64/-
	Traction force/kN	74.00	59.58
	Strain at both sides of the bar/με	71.47~1115.81 /116.63~1069.75	135.93 /−65.70
	The maximum load/kN	30.05	65.31
Initiation of shear crack	Strain at the midpoint of the bar/με	3240.16~2662.15 /2621.30~2327.46	2496.90~2018.67 /-
	Reduction of strain at the midpoint of the bar/με	578.01/293.84	478.23/-
	Strain at both sides of the bar/με	2071.82~2018.53 /2531.88~3405.52	1218.47/1189.96
	Reduction of strain at both sides of the bar /με	53.29/−873.64	-
	The maximum strain of the bar/με	4164.03 (c4)	2496.90 (s1)

**Table 2:** Impact results for SEN beams reinforced with CFRP bars.

Specimen Number	Impact Velocity (*v*)/mm/s	Impact Energy (*E_i_*)/J	Maximum of Reaction Force (*R*_max_)/kN	Maximum of Mid-Span Deflection (*δ*_max_) /mm	Absorbed Energy (*E_k_*)/J	Absorbed Energy Ratio (*E_k_*/*E_i_*)/%
F2-50-1	9.89 × 102	51.86	13.62	1.95	14.61	28.17
F2-50-2	9.89 × 102	51.86	14.81	2.01	13.98	26.96
F2-50-3	9.89 × 102	51.86	13.42	1.88	13.94	26.88
F2-150-1	1.71 × 103	155.05	23.90	3.12	35.52	22.91
F2-150-2	1.71 × 103	155.05	32.51	3.24	42.91	27.67
F2-150-3	1.71 × 103	155.05	31.02	3.07	39.02	25.17
F2-250-1	2.21 × 103	258.98	32.27	3.27	52.24	20.17
F2-250-2	2.21 × 103	258.98	33.62	3.47	54.22	20.94
F2-250-3	2.21 × 103	258.98	30.16	3.11	50.07	19.33

**Table 3:** Impact results for SEN beams reinforced with steel bars.

Specimen Number	Impact Velocity (*v*)/mm/s	Impact Energy (*E_i_*)/J	Maximum of Reaction Force (*R*_max_)/kN	Maximum of Mid-Span Deflection (*δ*_max_)/mm	Absorbed Energy (*E_k_*)/J	Absorbed Energy Ratio (*E_k_*/*E_i_*)/%
S2-50-1	9.89 × 102	51.86	24.20	1.49	17.57	33.88
S2-50-2	9.89 × 102	51.86	24.00	1.48	14.30	27.57
S2-50-3	9.89 × 102	51.86	19.61	1.36	15.80	30.47
S2-150-1	1.71 × 103	155.05	51.02	2.61	31.07	20.04
S2-150-2	1.71 × 103	155.05	45.80	2.54	40.71	26.26
S2-150-3	1.71 × 103	155.05	43.41	2.47	39.53	25.50
S2-250-1	2.21 × 103	258.98	53.13	3.16	65.95	25.47
S2-250-2	2.21 × 103	258.98	49.8	3.04	60.68	23.43
S2-250-3	2.21 × 103	258.98	63.6	3.44	80.57	31.11

## Data Availability

The data presented in this study are available on request from the corresponding author. The data are not publicly available due to some of the data pertaining to unpublished papers.
